# Supplementary material for: Optimization of Fermentation Conditions for Enhanced Single Cell Protein Production by Rossellomorea marisflavi NDS and Nutritional Composition Analysis
Source: Foods. 2025 Aug 30;14(17):3066. doi: 10.3390/foods14173066 (PMC12428340; doi:10.3390/foods14173066)
Supplement: Supplementary file 1 [file foods-14-03066-s001.zip › foods-3832694-supplementary.pdf]

Supplementary Figures

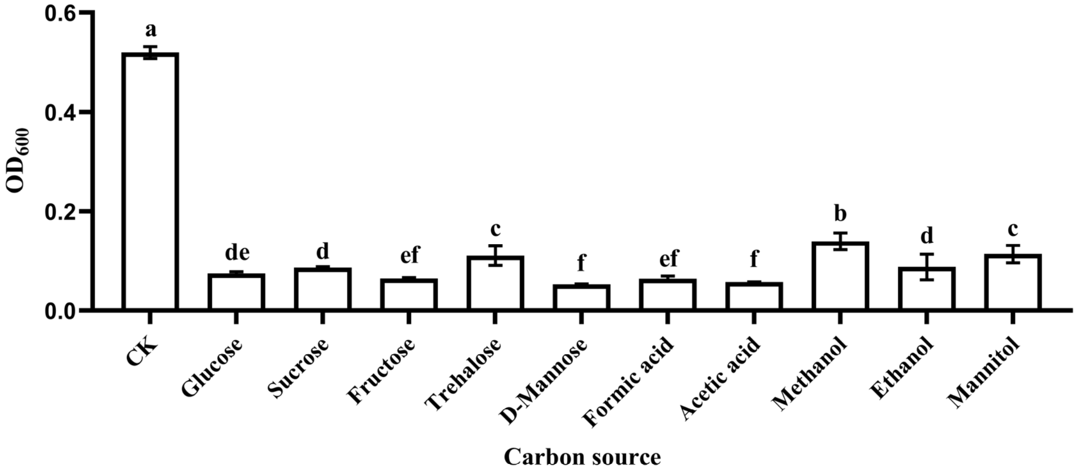

Figure S1. Effect of carbon sources on growth of *R. marisflavi* NDS. Growth was monitored by measuring optical density at OD_{600}. Data points represent the mean ± SD of three independent replicates (n=3). Mean values labeled with different letter are significantly different ($p < 0.05$).

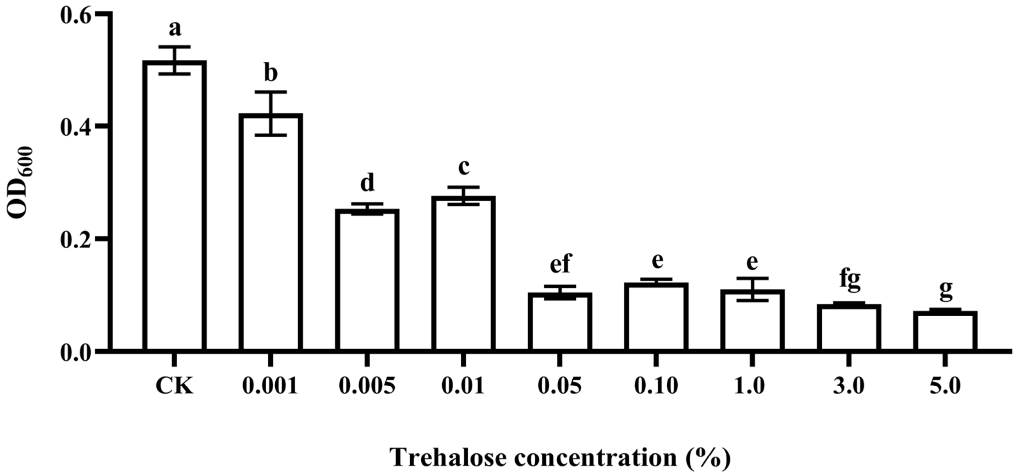

Figure S2. Effect of trehalose concentration on growth of *R. marisflavi* NDS. Growth was monitored by measuring optical density at OD_{600}. Data points represent the mean ± SD of three independent replicates (n=3). Mean values labeled with different letter

are significantly different ($p < 0.05$).

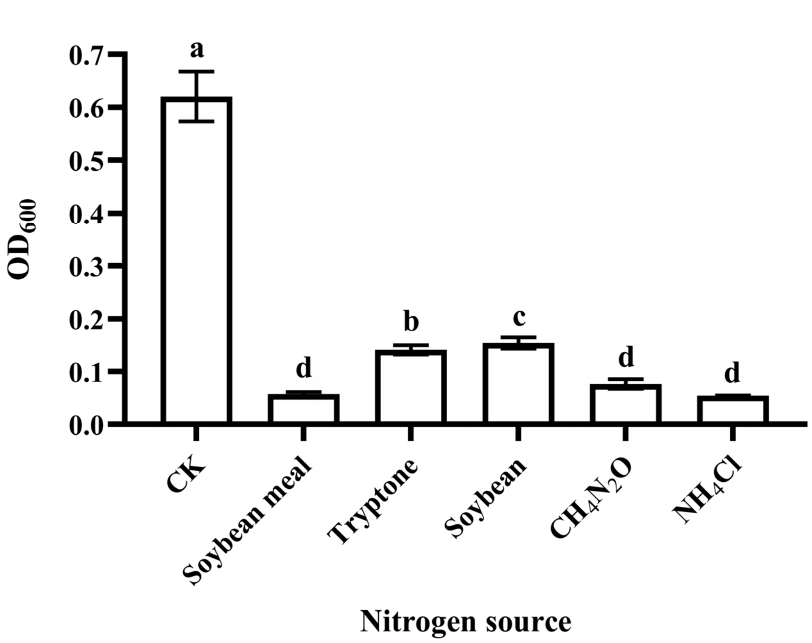

Figure S3. Effect of nitrogen sources on growth of *R. marisflavi* NDS. Growth was monitored by measuring optical density at OD_{600}. Data points represent the mean ± SD of three independent replicates (n=3). Mean values labeled with different letter are significantly different ($p < 0.05$).
